# Supplementary material for: Community factors and excess mortality in first wave of the COVID-19 pandemic in England
Source: Nat Commun. 2021 Jun 18;12:3755. doi: 10.1038/s41467-021-23935-x (PMC8213785; doi:10.1038/s41467-021-23935-x)
Supplement: Supplementary file 1 — Supplementary Information [file 41467_2021_23935_MOESM1_ESM.pdf]

## Supplementary information

# Community factors and excess mortality in first wave of the COVID-19 pandemic

Bethan Davies, Brandon L Parkes, James Bennett, Daniela Fecht,

Marta Blangiardo, Majid Ezzati, Paul Elliott

**Supplementary Table 1. Characteristics of the 6,791 Middle Super Output Areas in England.**

| | Mean | Median | Range | Inter-quartile range |
|---|---|---|---|---|
| Population | 8,288 | 7,985 | 2,224 – 26,513 | 6,831 – 9,320 |
| Population (M) | 4,098 | 3,926 | 1,089 – 14,535 | 3,348 – 4,629 |
| Population (F) | 4,191 | 4,052 | 1,135 – 11,978 | 3,468 – 4,722 |
| Population over 40 (M) | 1,994 | 1,926 | 410 – 4,459 | 1,660 – 2,277 |
| Population over 40 (F) | 2,155 | 2,090 | 286 – 4,794 | 1,788 – 2,155 |
| Area ($km^2$) | 19.2 | 3.04 | 0.294 – 1128 | 1.69 – 10.5 |
| Baseline deaths (M), per 100,000 males over 40, 2015 – 2019 | 447 | 438 | 53.7 – 1,200 | 356 – 529 |
| Baseline deaths (F), per 100,000 females over 40, 2015 – 2019 | 427 | 406 | 0 – 1,502 | 313 – 519 |

M – male; F – female; km- kilometre.

**Supplementary Table 2. Characteristics of the study and comparison populations.**

| | Comparison period (1 March – 31 May, 2015 – 2019) | Study period (1 March – 31 May 2020) | | |
|---|---|---|---|---|
| | **Mean deaths per year (%)** | **Total deaths (%)** | **Deaths with COVID-19 as underlying cause of death (%)** | **Deaths with COVID-19 mentioned on death certificate (%)** |
| **Total** | 121,441 | 174,327 | 42,000 | 44,913 |
| **Sex** | | | | |
| Male | 59,397 (48.9) | 88,092(50.5) | 23,349 (55.6) | 24,917 (55.5) |
| Female | 62,044 (51.1) | 86,235 (49.5) | 18,651 (44.4) | 19,996 (44.5) |
| **Age** | | | | |
| 40-59 | 10,329 (8.51) | 12,963(7.44) | 2,523 (6.01) | 2,755 (6.13) |
| 60-69 | 14,300 (11.8) | 18,286 (10.5) | 4,110 (9.79) | 4,422 (9.85) |
| 70-79 | 26,976 (22.2) | 38,988 (22.4) | 9,470 (22.5) | 10,188 (22.7) |
| 80+ | 69,835 (57.5) | 104,090 (59.7) | 25,897 (61.7) | 27,548 (61.3) |
| **Place of death** | | | | |
| Hospital | 56,780 (46.8) | 70,188 (40.3) | 26,890 (64.0) | 28,578 (63.6) |
| Care home | 27,128 (22.3) | 52,268 (30.0) | 12,611 (30.0) | 13,301 (29.6) |
| Home | 28,316 (23.3) | 42,004(24.1) | 1,791 (4.26) | 2,046 (4.56) |
| Hospice | 6,908 (5.69) | 6,402 (3.67) | 382 (0.91) | 620 (1.38) |
| Other/elsewhere | 2,309 (1.89) | 3,465 (1.80) | 326 (0.77) | 368 (0.81) |
| **Population on income support (%)** | | | | |
| ≥ 0.80 to < 6.00 | 22,893 (18.9) | 33,211 (19.1) | 7,736 (18.4) | 8,286 (18.4) |
| ≥ 6.00 to < 8.79 | 25,060 (20.6) | 34,859 (20.0) | 7,629 (18.2) | 8,185 (18.2) |
| ≥ 8.79 to < 12.7 | 25,287 (20.8) | 35,615 (20.4) | 8,146 (19.4) | 8,739 (19.5) |
| ≥ 12.7 to < 18.8 | 24,449 (20.1) | 35,238 (20.2) | 9,090 (21.6) | 9,673 (21.5) |
| ≥ 18.8 to < 48.9 | 23,750 (19.6) | 35,404 (20.3) | 9,399 (22.4) | 10,030 (22.3) |
| **Population density (population per km$^2$)** | | | | |
| ≥ 5.65 to < 456 | 26,586 (21.9) | 35,566 (20.4) | 6,668 (15.9) | 7,140 (15.9) |
| ≥ 456 to < 1,877 | 27,230 (22.4) | 38,145 (21.9) | 8,558 (20.4) | 9,157 (20.4) |
| ≥ 1,877 to < 3,445 | 25,443 (21.0) | 36,171 (20.7) | 8,654 (20.6) | 9,273 (20.6) |
| ≥ 3,445 to < 5,340 | 24,263 (20.0) | 35,550 (20.4) | 9,071 (21.6) | 9,728 (21.7) |
| ≥ 5,340 to < 29,000 | 17,917 (14.8) | 28,895 (16.6) | 9,049 (21.5) | 9,615 (21.4) |
| **Population non-White (%)** | | | | |
| ≥ 0.43 to < 2.10 | 28,389 (23.4) | 36,172 (21.3) | 6,924 (16.5) | 7,402 (16.5) |

| | | | | |
|---|---|---|---|---|
| ≥ 2.10 to < 3.72 | 27,426 (22.6) | 37,588 (21.6) | 7,996 (19.0) | 8,610 (19.2) |
| ≥ 3.72 to < 8.28 | 25,531 (21.0) | 35,505 (20.4) | 8,082 (19.2) | 8,647 (19.3) |
| ≥ 8.28 to < 22.2 | 22,418 (18.5) | 33,559 (19.3) | 8,769 (20.9) | 9,380 (20.9) |
| ≥ 22.2 to < 94.4 | 17,677 (14.6) | 30,503 (17.5) | 10,229 (24.4) | 10,874 (24.2) |
| **Overcrowded homes (%)** | | | | |
| ≥ 0.334 to < 1.65 | 26,440 (21.8) | 36,047 (20.7) | 7,353 (17.5) | 7,904 (17.6) |
| ≥ 1.65 to < 2.42 | 26,783 (22.1) | 36,753 (21.1) | 7,776 (18.5) | 8,355 (18.6) |
| ≥ 2.42 to < 3.67 | 26,578 (21.9) | 36,955 (21.2) | 8,394 (20.0) | 8,975 (20.0) |
| ≥ 3.67 to < 6.26 | 23,998 (19.8) | 34,687 (19.9) | 8,661 (20.6) | 9,265 (20.6) |
| ≥ 6.26 to < 36.5 | 17,641 (14.5) | 29,885 (17.1) | 9,816 (23.4) | 10,414 (23.2) |
| **$NO_2$ annual average (μg/$m^3$)** | | | | |
| ≥ 3.60 to < 9.52 | 28,654 (23.6) | 37,311 (21.4) | 6,414 (15.3) | 6,900 (15.4) |
| ≥ 9.52 to < 12.3 | 26,850 (22.1) | 36,923 (21.2) | 7,931 (18.9) | 8,493 (18.9) |
| ≥ 12.3 to < 14.9 | 24,460 (20.1) | 34,569 (19.8) | 8,145 (19.4) | 8,747 (19.5) |
| ≥ 14.9 to < 18.7 | 23,904 (19.7) | 35,502 (20.4) | 9,456 (22.5) | 10,101 (22.5) |
| ≥ 18.7 to < 47.5 | 17,571 (14.5) | 30,022 (17.2) | 10,054 (23.9) | 10,672 (23.8) |
| **$PM_{2.5}$ annual average (μg/$m^3$)** | | | | |
| ≥ 5.05 to < 7.76 | 27,675 (22.8) | 37,213 (21.3) | 7,760 (18.5) | 8,332 (18.6) |
| ≥ 7.76 to < 8.91 | 26,197 (21.6) | 36,485 (20.9) | 8,293 (19.7) | 8,852 (19.7) |
| ≥ 8.91 to < 9.88 | 26,148 (21.5) | 36,075 (20.7) | 7,774 (18.5) | 8,382 (18.7) |
| ≥ 9.88 to < 10.9 | 23,720 (19.5) | 34,519 (19.8) | 8,239 (19.6) | 8,803 (19.6) |
| ≥ 10.9 to < 14.4 | 17,700 (14.6) | 30,035 (17.2) | 9,934 (23.7) | 10,544 (23.5) |
| **Care homes per 1,000 population** | | | | |
| 0 | 19,472 (16.0) | 27,122 (15.6) | 6,703 (16.0) | 7,196 (16.0) |
| ≥ 0.040 to < 0.145 | 19,445 (16.0) | 28,106 (16.1) | 7,013 (16.7) | 7,537 (16.8) |
| ≥ 0.145 to < 0.265 | 24,181 (19.9) | 35,164 (20.2) | 8,769 (20.9) | 9,340 (20.8) |
| ≥ 0.265 to < 0.438 | 27,244 (22.4) | 39,027 (22.4) | 9,203 (21.9) | 9,793 (21.8) |
| ≥ 0.438 to < 4.23 | 31,098 (25.6) | 44,908 (25.8) | 10,312 (24.6) | 11,047 (24.6) |

**Supplementary Table 3. Excess death rates, percent increase in mortality and posterior probabilities for Middle Super Output Areas in England.**

| | Mean | Median | Range | Inter-quartile range |
|---|---|---|---|---|
| Excess death rate (M), per 100,000 males over 40 | 200 | 174 | -337 – 1482 | 88.1 – 283 |
| Excess death rate (F), per 100,000 females over 40 | 163 | 131 | -240 - 1662 | 48.1 - 235 |
| Percent increase in mortality (M) | 48.0 | 42.0 | -38.0 – 249 | 21.8 – 67.5 |
| Percent increase in mortality (F) | 40.9 | 34.9 | -42.4 - 333 | 14.8 – 60.0 |
| Posterior probability that excess rate is greater than zero (M) | 0.844 | 0.917 | 0.034 – 1.00 | 0.767 – 0.982 |
| Posterior probability that excess rate is greater than zero (F) | 0.776 | 0.845 | 0.012 – 1.00 | 0.651 – 0.953 |

M – male; F – female.

**Supplementary Table 4. One-variable-at-a-time models for community characteristics.** Age is included in all the models.

| Variable | n-tile | Males | | Females | |
|---|---|---|---|---|---|
| | | **Mean** | **95% credible interval** | **Mean** | **95% credible interval** |
| **Population on income support** | 1 (lowest) | ref | ref | ref | ref |
| | 2 | 1.04 | 1.01 – 1.08 | 1.00 | 0.97 – 1.03 |
| | 3 | 1.10 | 1.07 – 1.13 | 1.04 | 1.00 – 1.07 |
| | 4 | 1.15 | 1.12 – 1.19 | 1.07 | 1.03 – 1.10 |
| | 5 (highest) | 1.23 | 1.19 – 1.27 | 1.16 | 1.12 – 1.20 |
| | % of variation contributed by variable | 7.49 | 5.48 – 9.72 | 4.05 | 2.64 – 5.69 |
| **Population density** | 1 (lowest) | ref | ref | ref | ref |
| | 2 | 1.06 | 1.03 – 1.10 | 1.04 | 1.01 – 1.07 |
| | 3 | 1.10 | 1.07 – 1.14 | 1.05 | 1.01 – 1.08 |
| | 4 | 1.11 | 1.07 – 1.14 | 1.10 | 1.06 – 1.14 |
| | 5 (highest) | 1.16 | 1.12 – 1.21 | 1.12 | 1.07 – 1.16 |
| | % of variation contributed by variable | 3.67 | 2.13 – 5.47 | 2.06 | 1.01 – 3.38 |
| **Population non-White** | 1 (lowest) | ref | ref | ref | ref |
| | 2 | 1.04 | 1.01 – 1.07 | 1.05 | 1.01 – 1.08 |
| | 3 | 1.07 | 1.04 – 1.10 | 1.05 | 1.02 – 1.09 |
| | 4 | 1.12 | 1.09 – 1.16 | 1.12 | 1.08 – 1.16 |
| | 5 (highest) | 1.26 | 1.21 – 1.32 | 1.21 | 1.16 – 1.26 |
| | % of variation contributed by variable | 9.56 | 6.59 – 12.9 | 5.59 | 3.40 – 8.14 |
| **Overcrowded homes** | 1 (lowest) | ref | ref | ref | ref |
| | 2 | 1.05 | 1.02 – 1.07 | 1.02 | 0.99 – 1.06 |
| | 3 | 1.09 | 1.06 – 1.12 | 1.06 | 1.03 – 1.09 |
| | 4 | 1.16 | 1.13 – 1.20 | 1.10 | 1.07 – 1.14 |
| | 5 (highest) | 1.28 | 1.23 – 1.32 | 1.19 | 1.15 – 1.24 |
| | % of variation contributed by variable | 10.45 | 7.69 – 13.49 | 4.89 | 3.05 – 6.98 |
| **$NO_2$** | 1 (lowest) | ref | ref | ref | ref |
| | 2 | 1.05 | 1.01 – 1.08 | 1.04 | 1.00 – 1.07 |
| | 3 | 1.09 | 1.06 – 1.13 | 1.04 | 1.01 – 1.08 |
| | 4 | 1.14 | 1.09 – 1.18 | 1.12 | 1.07 – 1.16 |
| | 5 (highest) | 1.22 | 1.16 – 1.28 | 1.17 | 1.12 – 1.23 |
| | % of variation contributed by variable | 6.90 | 4.07 – 10.24 | 4.21 | 2.19 – 6.68 |
| | 1 (lowest) | ref | ref | ref | ref |

| | | | | | |
|---|---|---|---|---|---|
| **$PM_{2.5}$** | 2 | 1.06 | 1.02 – 1.11 | 1.04 | 1.00 – 1.08 |
| | 3 | 1.13 | 1.07 – 1.18 | 1.06 | 1.01 – 1.12 |
| | 4 | 1.18 | 1.11 – 1.24 | 1.10 | 1.04 – 1.17 |
| | 5 (highest) | 1.29 | 1.21 – 1.38 | 1.19 | 1.11 – 1.28 |
| | % of variation contributed by variable | 11.2 | 6.01 – 17.4 | 4.77 | 1.83 – 8.69 |
| **Care home per 1,000 population** | 1 (lowest) | ref | ref | ref | ref |
| | 2 | 1.10 | 1.06 – 1.13 | 1.13 | 1.09 – 1.16 |
| | 3 | 1.12 | 1.09 – 1.15 | 1.18 | 1.14 – 1.22 |
| | 4 | 1.14 | 1.11 – 1.18 | 1.21 | 1.18 – 1.25 |
| | 5 (highest) | 1.21 | 1.17 – 1.24 | 1.27 | 1.22 – 1.31 |
| | % of variation contributed by variable | 5.95 | 4.28 – 7.82 | 8.76 | 6.62 – 11.09 |

$NO_2$ – nitrogen dioxide; PM2.5 – particulate matter 2.5 μ diameter

**Supplementary Table 5. Correlation between community characteristics for Middle Super Output Areas in England:** Kendall's Tau coefficients between covariates (quintiles), n = 6,791 MSOAs.

| | **Population on income support** | **Population density** | **Non-White population** | **Overcrowding** | **$NO_2$** | **$PM_{2.5}$** | **Care homes per 1000 population*** |
|---|---|---|---|---|---|---|---|
| **Population on income support** | 1 | | | | | | |
| **Population density** | 0.37 | 1 | | | | | |
| **Population non-White** | 0.21 | 0.56 | 1 | | | | |
| **Overcrowding** | 0.55 | 0.61 | 0.61 | 1 | | | |
| **$NO_2$** | 0.26 | 0.59 | 0.67 | 0.56 | 1 | | |
| **$PM_{2.5}$** | 0.04 | 0.39 | 0.55 | 0.42 | 0.55 | 1 | |
| **Care homes per 1000 population*** | -0.04 | -0.17 | -0.17 | -0.16 | -0.20 | -0.16 | 1 |

*All MSOAs with a care home density of 0 (n=1,535 MSOAs) are in quintile 1. Quintile 2 has reduced number of MSOAs (n=1,182 MSOAs)

**Supplementary Table 6. Mutually adjusted multivariable models for all MSOA characteristics.** Age is included in all the models.

| **Variable** | **Quintile** | **Males** | | **Females** | |
|---|---|---|---|---|---|
| | | **Mean** | **95% credible interval** | **Mean** | **95% credible interval** |
| **Population on income support** | 1 (lowest) | ref | ref | ref | ref |
| | 2 | 1.02 | 0.99 – 1.05 | 0.97 | 0.94 – 1.01 |
| | 3 | 1.04 | 1.01 – 1.08 | 1.00 | 0.96 – 1.03 |
| | 4 | 1.07 | 1.03 – 1.12 | 1.02 | 0.97 – 1.06 |
| | 5 (highest) | 1.11 | 1.06 – 1.17 | 1.09 | 1.03 – 1.14 |
| **Population density** | 1 (lowest) | ref | ref | ref | ref |
| | 2 | 1.02 | 0.99 – 1.05 | 1.01 | 0.98 – 1.05 |
| | 3 | 1.02 | 0.98 – 1.06 | 1.00 | 0.96 – 1.04 |
| | 4 | 0.99 | 0.95 – 1.03 | 1.03 | 0.98 – 1.07 |
| | 5 (highest) | 0.99 | 0.95 – 1.04 | 1.00 | 0.95 – 1.06 |
| **Population non-White** | 1 (lowest) | ref | ref | ref | ref |
| | 2 | 1.01 | 0.98 – 1.04 | 1.03 | 1.00 – 1.07 |
| | 3 | 1.02 | 0.99 – 1.06 | 1.02 | 0.98 – 1.06 |
| | 4 | 1.03 | 0.99 – 1.08 | 1.06 | 1.01 – 1.12 |
| | 5 (highest) | 1.08 | 1.02 – 1.15 | 1.09 | 1.02 – 1.16 |
| **Overcrowded homes** | 1 (lowest) | ref | ref | ref | ref |
| | 2 | 1.02 | 0.99 – 1.05 | 1.01 | 0.98 – 1.05 |
| | 3 | 1.03 | 0.99 – 1.07 | 1.02 | 0.97 – 1.06 |
| | 4 | 1.07 | 1.02 – 1.12 | 1.02 | 0.97 – 1.07 |
| | 5 (highest) | 1.12 | 1.05 – 1.20 | 1.06 | 0.99 – 1.13 |
| **$NO_2$** | 1 (lowest) | ref | ref | ref | ref |
| | 2 | 1.01 | 0.97 – 1.05 | 1.02 | 0.98 – 1.05 |
| | 3 | 1.02 | 0.97 – 1.07 | 1.00 | 0.95 – 1.05 |
| | 4 | 1.00 | 0.95 – 1.06 | 1.03 | 0.97 – 1.10 |
| | 5 (highest) | 1.02 | 0.95 – 1.09 | 1.05 | 0.97 – 1.12 |
| **$PM_{2.5}$** | 1 (lowest) | ref | ref | ref | ref |
| | 2 | 1.00 | 0.96 – 1.05 | 0.99 | 0.95 – 1.04 |
| | 3 | 1.03 | 0.97 – 1.09 | 0.98 | 0.92 – 1.04 |
| | 4 | 1.02 | 0.95 – 1.10 | 0.98 | 0.91 – 1.05 |
| | 5 (highest) | 1.07 | 0.98 – 1.17 | 1.02 | 0.93 – 1.11 |
| **Care homes per 1,000 population** | 1 (lowest | ref | ref | ref | ref |
| | 2 | 1.09 | 1.06 – 1.12 | 1.12 | 1.08 – 1.16 |
| | 3 | 1.13 | 1.09 – 1.16 | 1.19 | 1.15 – 1.22 |
| | 4 | 1.15 | 1.12 – 1.18 | 1.22 | 1.19 – 1.26 |
| | 5 (highest) | 1.21 | 1.18 – 1.25 | 1.27 | 1.23 – 1.31 |
| % of variation contributed by all variables | | 18.2 | 15.0 – 21.8 | 15.3 | 12.8 – 18.2 |
| % of variance explained by local clustering for model with all variables | | 31.4 | 28.7 – 34.2 | 19.2 | 17.2 – 21.3 |
| % of variance explained by local clustering for model with no variables | | 40.4 | 37.6 – 42.4 | 22.3 | 20.5 – 23.9 |

**Supplementary Table 7. Mutually adjusted multivariable models for all MSOA characteristics, results of sensitivity analyses.** Age is included in all the models.

**a) Penalised complexity prior.**

| Variable | Quintile | Males | | Females | |
|---|---|---|---|---|---|
| | | **Mean** | **95% credible interval** | **Mean** | **95% credible interval** |
| **Population on income support** | 1 (lowest) | ref | ref | ref | ref |
| | 2 | 1.02 | 0.99 – 1.05 | 0.97 | 0.94 – 1.00 |
| | 3 | 1.04 | 1.01 – 1.08 | 0.99 | 0.96 – 1.03 |
| | 4 | 1.07 | 1.03 – 1.12 | 1.00 | 0.97 – 1.06 |
| | 5 (highest) | 1.11 | 1.06 – 1.16 | 1.09 | 1.03 – 1.14 |
| **Population density** | 1 (lowest) | ref | ref | ref | ref |
| | 2 | 1.02 | 0.99 – 1.05 | 1.01 | 0.98 – 1.05 |
| | 3 | 1.01 | 0.98 – 1.06 | 1.00 | 0.96 – 1.04 |
| | 4 | 0.99 | 0.96 – 1.03 | 1.03 | 0.98 – 1.07 |
| | 5 (highest) | 0.99 | 0.95 – 1.04 | 1.00 | 0.95 – 1.05 |
| **Population non-White** | 1 (lowest) | ref | ref | ref | ref |
| | 2 | 1.01 | 0.98 – 1.04 | 1.03 | 1.00 – 1.07 |
| | 3 | 1.02 | 0.99 – 1.06 | 1.02 | 0.98 – 1.06 |
| | 4 | 1.03 | 0.99 – 1.08 | 1.07 | 1.02 – 1.12 |
| | 5 (highest) | 1.08 | 1.02 – 1.15 | 1.10 | 1.03 – 1.17 |
| **Overcrowded homes** | 1 (lowest) | ref | ref | ref | ref |
| | 2 | 1.02 | 0.99 – 1.05 | 1.01 | 0.98 – 1.05 |
| | 3 | 1.03 | 0.99 – 1.07 | 1.02 | 0.98 – 1.06 |
| | 4 | 1.07 | 1.02 – 1.12 | 1.02 | 0.97 – 1.07 |
| | 5 (highest) | 1.12 | 1.05 – 1.20 | 1.06 | 0.99 – 1.14 |
| **$NO_2$** | 1 (lowest) | ref | ref | ref | ref |
| | 2 | 1.01 | 0.97 – 1.05 | 1.02 | 0.98 – 1.06 |
| | 3 | 1.02 | 0.98 – 1.07 | 1.00 | 0.96 – 1.05 |
| | 4 | 1.01 | 0.95 – 1.06 | 1.03 | 0.98 – 1.09 |
| | 5 (highest) | 1.02 | 0.95 – 1.09 | 1.05 | 0.98 – 1.13 |
| **$PM_{2.5}$** | 1 (lowest) | ref | ref | ref | ref |
| | 2 | 1.00 | 0.96 – 1.05 | 0.99 | 0.95 – 1.04 |
| | 3 | 1.03 | 0.97 – 1.09 | 0.98 | 0.93 – 1.04 |
| | 4 | 1.02 | 0.95 – 1.09 | 0.98 | 0.91 – 1.05 |
| | 5 (highest) | 1.07 | 0.98 – 1.17 | 1.02 | 0.93 – 1.12 |
| **Care homes per 1,000 population** | 1 (lowest | ref | ref | ref | ref |
| | 2 | 1.09 | 1.06 – 1.12 | 1.12 | 1.08 – 1.16 |
| | 3 | 1.13 | 1.10 – 1.17 | 1.18 | 1.15 – 1.22 |
| | 4 | 1.15 | 1.12 – 1.18 | 1.22 | 1.18 – 1.26 |
| | 5 (highest) | 1.21 | 1.18 – 1.25 | 1.27 | 1.23 – 1.31 |
| % of variation contributed by all variables | | 18.8 | 15.0 – 23,3 | 15.9 | 12.9 – 19.2 |

**b) Priors on the hyperparameters $\tau_v$ and $\tau_u$: logGamma(0.5, 0.05).**

| Variable | Quintile | Males | | Females | |
|---|---|---|---|---|---|
| | | **Mean** | **95% credible interval** | **Mean** | **95% credible interval** |
| **Population on income support** | 1 (lowest) | ref | ref | ref | ref |
| | 2 | 1.02 | 0.99 – 1.05 | 0.97 | 0.94 – 1.00 |
| | 3 | 1.04 | 1.01 – 1.08 | 0.99 | 0.96 – 1.03 |
| | 4 | 1.07 | 1.03 – 1.12 | 1.01 | 0.97 – 1.06 |
| | 5 (highest) | 1.11 | 1.06 – 1.17 | 1.09 | 1.03 – 1.14 |
| **Population density** | 1 (lowest) | ref | ref | ref | ref |
| | 2 | 1.02 | 0.99 – 1.05 | 1.01 | 0.98 – 1.05 |
| | 3 | 1.01 | 0.98 – 1.06 | 1.00 | 0.96 – 1.03 |
| | 4 | 0.99 | 0.95 – 1.03 | 1.03 | 0.98 – 1.07 |
| | 5 (highest) | 0.99 | 0.95 – 1.04 | 1.00 | 0.95 – 1.05 |
| **Population non-White** | 1 (lowest) | ref | ref | ref | ref |
| | 2 | 1.01 | 0.98 – 1.04 | 1.03 | 1.00 – 1.07 |
| | 3 | 1.02 | 0.99 – 1.06 | 1.02 | 0.98 – 1.06 |
| | 4 | 1.03 | 0.99 – 1.08 | 1.06 | 1.01 – 1.12 |
| | 5 (highest) | 1.08 | 1.02 – 1.15 | 1.09 | 1.03 – 1.16 |
| **Overcrowded homes** | 1 (lowest) | ref | ref | ref | ref |
| | 2 | 1.02 | 0.99 – 1.05 | 1.01 | 0.98 – 1.05 |
| | 3 | 1.03 | 0.99 – 1.07 | 1.02 | 0.98 – 1.06 |
| | 4 | 1.07 | 1.02 – 1.12 | 1.02 | 0.97 – 1.08 |
| | 5 (highest) | 1.12 | 1.05 – 1.20 | 1.06 | 0.99 – 1.14 |
| **$NO_2$** | 1 (lowest) | ref | ref | ref | ref |
| | 2 | 1.01 | 0.97 – 1.04 | 1.02 | 0.98 – 1.05 |
| | 3 | 1.02 | 0.97 – 1.06 | 1.00 | 0.96 – 1.05 |
| | 4 | 1.00 | 0.95 – 1.06 | 1.03 | 0.97 – 1.09 |
| | 5 (highest) | 1.02 | 0.95 – 1.09 | 1.05 | 0.97 – 1.12 |
| **$PM_{2.5}$** | 1 (lowest) | ref | ref | ref | ref |
| | 2 | 1.00 | 0.96 – 1.05 | 0.99 | 0.95 – 1.04 |
| | 3 | 1.03 | 0.97 – 1.09 | 0.98 | 0.92 – 1.04 |
| | 4 | 1.02 | 0.95 – 1.10 | 0.98 | 0.91 – 1.05 |
| | 5 (highest) | 1.08 | 0.98 – 1.17 | 1.02 | 0.93 – 1.12 |
| **Care homes per 1,000 population** | 1 (lowest | ref | ref | ref | ref |
| | 2 | 1.09 | 1.06 – 1.12 | 1.12 | 1.08 – 1.16 |
| | 3 | 1.13 | 1.09 – 1.16 | 1.18 | 1.15 – 1.22 |
| | 4 | 1.15 | 1.12 – 1.18 | 1.22 | 1.19 – 1.26 |
| | 5 (highest) | 1.21 | 1.18 – 1.25 | 1.27 | 1.23 – 1.31 |
| % of variation contributed by all variables | | 18.6 | 14.8 – 23.1 | 15.8 | 12.8 – 19.0 |

**c) Excluding care homes per 1,000 population**

| Variables | Quintile | Males | | Females | |
|---|---|---|---|---|---|
| | | **Mean** | **95% credible interval** | **Mean** | **95% credible interval** |
| **Population on income support** | 1 (lowest) | ref | ref | ref | ref |
| | 2 | 1.03 | 1.00 – 1.06 | 0.98 | 0.95 – 1.02 |
| | 3 | 1.06 | 1.02 – 1.10 | 1.01 | 0.97 – 1.05 |
| | 4 | 1.09 | 1.05 – 1.14 | 1.03 | 0.99 – 1.08 |
| | 5 (highest) | 1.13 | 1.08 – 1.19 | 1.10 | 1.05 – 1.17 |
| **Population density** | 1 (lowest) | Ref | ref | ref | ref |
| | 2 | 1.02 | 0.99 – 1.05 | 1.01 | 0.98 – 1.05 |
| | 3 | 1.01 | 0.98 – 1.05 | 0.99 | 0.95 – 1.03 |
| | 4 | 0.98 | 0.94 – 1.03 | 1.01 | 0.97 – 1.06 |
| | 5 (highest) | 0.98 | 0.94 – 1.03 | 0.99 | 0.94 – 1.04 |
| **Population non-White** | 1 (lowest) | ref | Ref | ref | ref |
| | 2 | 1.02 | 0.99 – 1.05 | 1.04 | 1.00 – 1.07 |
| | 3 | 1.03 | 1.00 – 1.07 | 1.03 | 0.99 – 1.07 |
| | 4 | 1.05 | 1.00 – 1.11 | 1.08 | 1.03 – 1.13 |
| | 5 (highest) | 1.10 | 1.04 – 1.17 | 1.11 | 1.04 – 1.19 |
| **Overcrowded homes** | 1 (lowest) | ref | ref | ref | ref |
| | 2 | 1.02 | 0.98 – 1.05 | 1.01 | 0.98 – 1.05 |
| | 3 | 1.02 | 0.98 – 1.06 | 1.01 | 0.97 – 1.06 |
| | 4 | 1.06 | 1.01 – 1.11 | 1.01 | 0.96 – 1.06 |
| | 5 (highest) | 1.10 | 1.03 – 1.17 | 1.03 | 0.96 – 1.11 |
| **$NO_2$** | 1 (lowest) | ref | ref | ref | ref |
| | 2 | 1.01 | 0.97 – 1.04 | 1.02 | 0.98 – 1.05 |
| | 3 | 1.01 | 0.97 – 1.06 | 0.99 | 0.95 – 1.04 |
| | 4 | 1.00 | 0.95 – 1.06 | 1.03 | 0.97 – 1.09 |
| | 5 (highest) | 1.0 | 0.94 – 1.08 | 1.04 | 0.96 – 1.11 |
| **$PM_{2.5}$** | 1 (lowest) | ref | ref | ref | ref |
| | 2 | 1.00 | 0.96 – 1.05 | 0.99 | 0.95 – 1.03 |
| | 3 | 1.02 | 0.96 – 1.08 | 0.97 | 0.91 – 1.02 |
| | 4 | 1.00 | 0.93 – 1.07 | 0.96 | 0.89 – 1.03 |
| | 5 (highest) | 1.04 | 0.95 – 1.13 | 0.98 | 0.89 – 1.08 |
| % of variation contributed by all variables | | 13.7 | 10.1 – 17.9 | 8.46 | 5.91 – 11.4 |

**d) Excluding care homes per 1,000 population and deaths in care homes**

| Covariate | Quintile | Males | | Females | |
|---|---|---|---|---|---|
| | | Mean | 95% credible interval | Mean | 95% credible interval |
| **Population on income support** | 1 (lowest) | ref | ref | ref | ref |
| | 2 | 1.05 | 1.02 – 1.09 | 1.02 | 0.99 – 1.06 |
| | 3 | 1.09 | 1.05 – 1.13 | 1.08 | 1.04 – 1.13 |
| | 4 | 1.13 | 1.08 – 1.18 | 1.14 | 1.09 – 1.20 |
| | 5 (highest) | 1.19 | 1.14 – 1.25 | 1.25 | 1.18 – 1.32 |
| **Population density** | 1 (lowest) | ref | ref | ref | ref |
| | 2 | 1.01 | 0.98 – 1.05 | 1.03 | 0.99 – 1.06 |
| | 3 | 1.01 | 0.97 – 1.05 | 1.02 | 0.98 – 1.06 |
| | 4 | 0.97 | 0.93 – 1.01 | 1.03 | 0.98 – 1.08 |
| | 5 (highest) | 0.97 | 0.92 – 1.02 | 0.99 | 0.94 – 1.05 |
| **Population non-White** | 1 (lowest) | ref | ref | ref | ref |
| | 2 | 1.00 | 0.97 – 1.03 | 1.01 | 0.98 – 1.05 |
| | 3 | 0.99 | 0.96 – 1.03 | 0.98 | 0.94 – 1.02 |
| | 4 | 0.99 | 0.95 – 1.04 | 1.03 | 0.98 – 1.09 |
| | 5 (highest) | 1.06 | 0.99 – 1.13 | 1.05 | 0.98 – 1.12 |
| **Overcrowded homes** | 1 (lowest) | ref | ref | ref | ref |
| | 2 | 1.02 | 0.98 – 1.05 | 1.02 | 0.99 – 1.06 |
| | 3 | 1.03 | 0.99 – 1.07 | 1.00 | 0.96 – 1.04 |
| | 4 | 1.07 | 1.02 – 1.12 | 1.02 | 0.96 – 1.07 |
| | 5 (highest) | 1.12 | 1.04 – 1.19 | 1.07 | 0.99 – 1.15 |
| **$NO_2$** | 1 (lowest) | ref | ref | ref | ref |
| | 2 | 1.01 | 0.97 – 1.05 | 0.98 | 0.95 – 1.02 |
| | 3 | 1.02 | 0.97 – 1.07 | 0.97 | 0.92 – 1.02 |
| | 4 | 1.01 | 0.95 – 1.07 | 0.99 | 0.94 – 1.05 |
| | 5 (highest) | 1.04 | 0.97 – 1.11 | 1.02 | 0.94 – 1.10 |
| **$PM_{2.5}$** | 1 (lowest) | ref | ref | ref | ref |
| | 2 | 1.01 | 0.96 – 1.05 | 0.98 | 0.94 – 1.03 |
| | 3 | 1.02 | 0.96 – 1.08 | 0.96 | 0.90 – 1.02 |
| | 4 | 1.02 | 0.95 – 1.10 | 0.96 | 0.89 – 1.04 |
| | 5 (highest) | 1.06 | 0.96 – 1.16 | 0.98 | 0.88 – 1.08 |
| % of variation contributed by all variables | | 21.8 | 16.7 – 27.7 | 25.2 | 19.6 – 31.5 |

**e) Both sexes combined**

| Variables | Quintile | Mean | 95% credible interval |
|---|---|---|---|
| **Population on income support** | 1 (lowest) | ref | ref |
| | 2 | 0.99 | 0.97 – 1.02 |
| | 3 | 1.01 | 0.98 – 1.04 |
| | 4 | 1.03 | 1.00 – 1.06 |
| | 5 (highest) | 1.07 | 1.03 – 1.11 |
| **Population density** | 1 (lowest) | ref | ref |
| | 2 | 1.02 | 0.99 – 1.04 |
| | 3 | 1.01 | 0.98 – 1.04 |
| | 4 | 1.01 | 0.98 – 1.04 |
| | 5 (highest) | 1.00 | 0.96 – 1.04 |
| **Population non-White** | 1 (lowest) | ref | ref |
| | 2 | 1.02 | 0.99 – 1.04 |
| | 3 | 1.02 | 0.99 – 1.05 |
| | 4 | 1.05 | 1.01 – 1.09 |
| | 5 (highest) | 1.09 | 1.04 – 1.14 |
| **Overcrowded homes** | 1 (lowest) | ref | ref |
| | 2 | 1.01 | 0.98 – 1.03 |
| | 3 | 1.01 | 0.98 – 1.04 |
| | 4 | 1.02 | 0.98 – 1.06 |
| | 5 (highest) | 1.06 | 1.00 – 1.11 |
| **$NO_2$** | 1 (lowest) | ref | ref |
| | 2 | 1.01 | 0.98 – 1.04 |
| | 3 | 1.01 | 0.98 – 1.05 |
| | 4 | 1.02 | 0.97 – 1.06 |
| | 5 (highest) | 1.03 | 0.97 – 1.09 |
| **$PM_{2.5}$** | 1 (lowest) | ref | ref |
| | 2 | 1.00 | 0.96 – 1.03 |
| | 3 | 1.00 | 0.95 – 1.05 |
| | 4 | 0.99 | 0.94 – 1.05 |
| | 5 (highest) | 1.03 | 0.96 – 1.11 |
| **Care homes per 1,000 population** | 1 (lowest | ref | ref |
| | 2 | 1.09 | 1.06 – 1.12 |
| | 3 | 1.13 | 1.11 – 1.16 |
| | 4 | 1.16 | 1.13 – 1.18 |
| | 5 (highest) | 1.20 | 1.18 – 1.23 |
| % of variation contributed by all variables | | 14.3 | 11.6 – 17.5 |

**Supplementary Table 8. Pearson's product moment correlation, excess deaths across MSOAs by numbers of samples.**

**a) Males**

| **Stage1, Stage 2** | 20,20 | 50,50 | 50,100 | 100,100 |
|---|---|---|---|---|
| 50,50 | 0.99495 | | | |
| 50,100 | 0.99491 | 0.99995 | | |
| 100,100 | 0.99329 | 0.99842 | 0.99847 | |
| 200,200 | 0.99246 | 0.99764 | 0.99758 | 0.99922 |

**b) Females**

| **Stage1, Stage 2** | 20,20 | 50,50 | 50,100 | 100,100 |
|---|---|---|---|---|
| 50,50 | 0.99494 | | | |
| 50,100 | 0.99491 | 0.99996 | | |
| 100,100 | 0.99354 | 0.99857 | 0.99862 | |
| 200,200 | 0.99275 | 0.99777 | 0.99782 | 0.99926 |

**Supplementary Table 9. Mean absolute difference in excess deaths across MSOAs by numbers of samples.**

a) **Males**

| **Stage1, Stage 2** | 20,20 | 50,50 | 50,100 | 100,100 |
|---|---|---|---|---|
| 50,50 | 0.240 | | | |
| 50,100 | 0.241 | 0.0244 | | |
| 100,100 | 0.276 | 0.135 | 0.133 | |
| 200,200 | 0.294 | 0.165 | 0.163 | 0.0941 |

b) **Females**

| **Stage1, Stage 2** | 20,20 | 50,50 | 50,100 | 100,100 |
|---|---|---|---|---|
| 50,50 | 0.277 | | | |
| 50,100 | 0.278 | 0.0262 | | |
| 100,100 | 0.314 | 0.150 | 0.147 | |
| 200,200 | 0.334 | 0.187 | 0.185 | 0.107 |

**Supplementary Fig. 1. Scatter plot showing male vs female percent increase in deaths age 40 and over for 6,701 Middle Super Output Areas in England.** Spearman's rank correlation coefficient 0.44

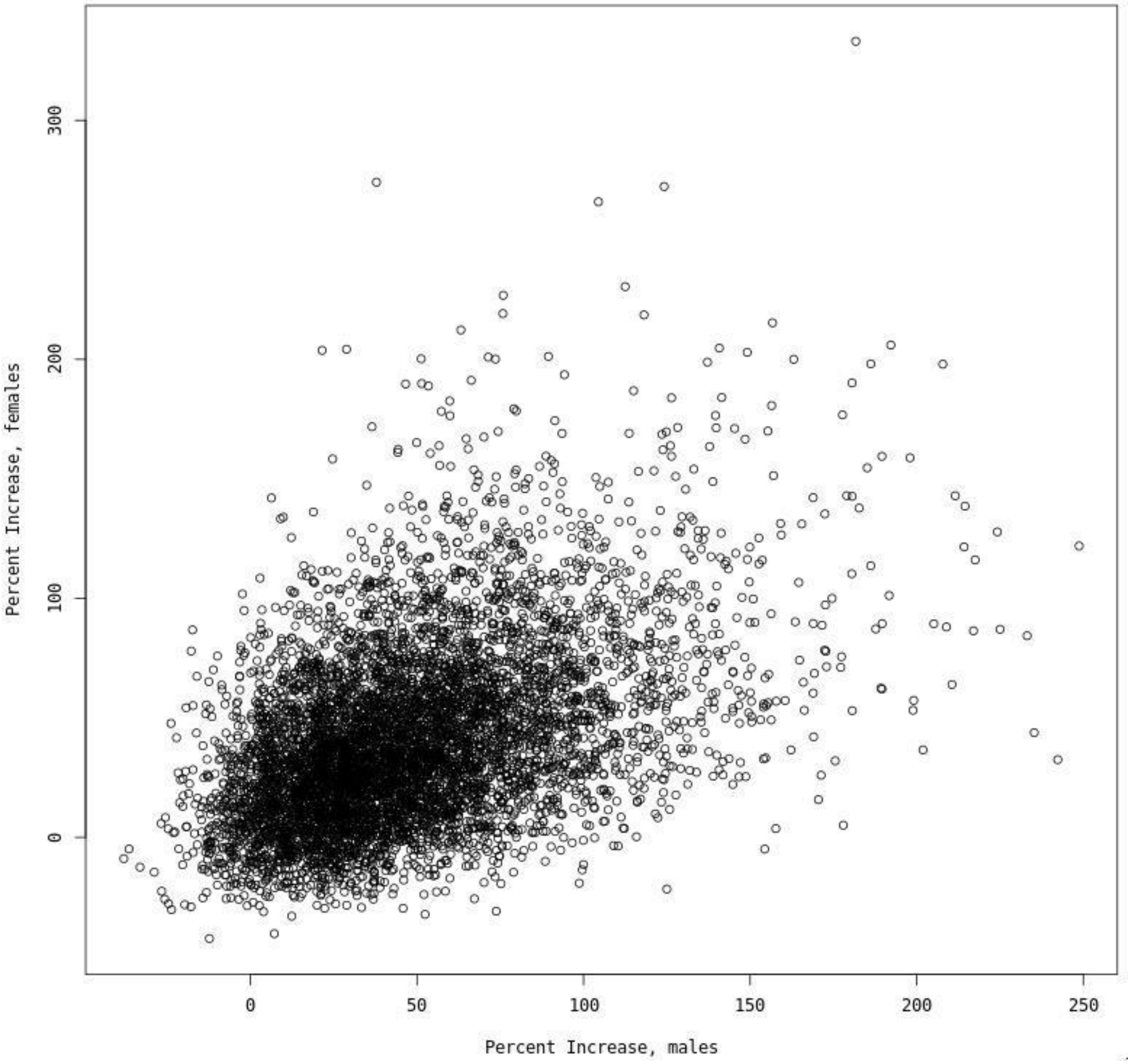

**Supplementary Fig. 2. Scatter plot showing male vs female excess mortality rate per 100,000 males/females age 40 and over for 6,701 Middle Super Output Areas in England.** Spearman's rank correlation coefficient 0.45

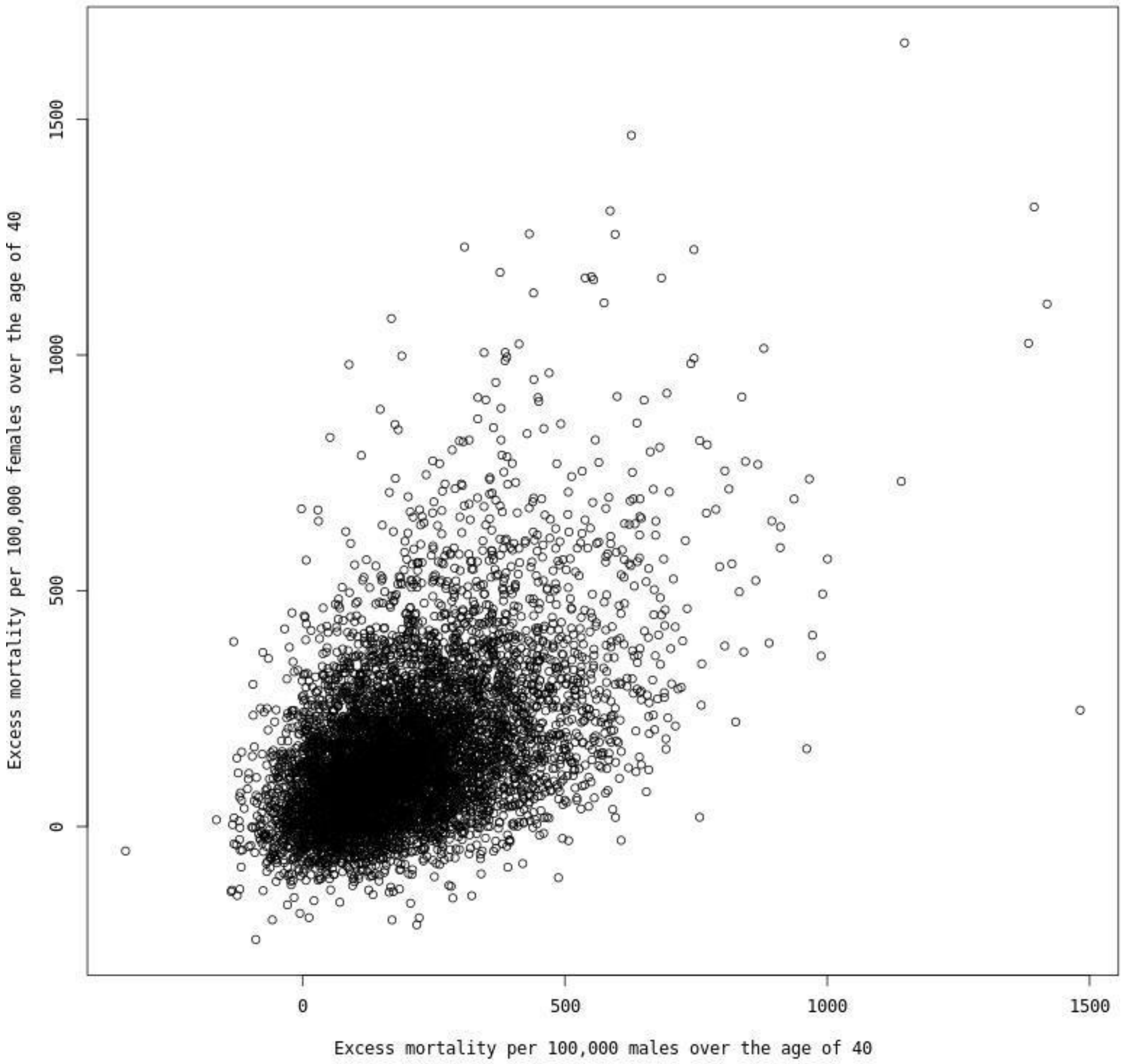

**Supplementary Fig. 3. Plot of the excess mortality rate (excess deaths per 100,000 population over the age of 40, both sexes combined) against the sero-prevalence rates from REACT 2 study (round 1, June 20th – July 13th 2020, Ward et al., 2021[1]) at Lower Tier Local Authority level (n = 317).** Spearman's rank correlation 0.501. R-squared 0.391

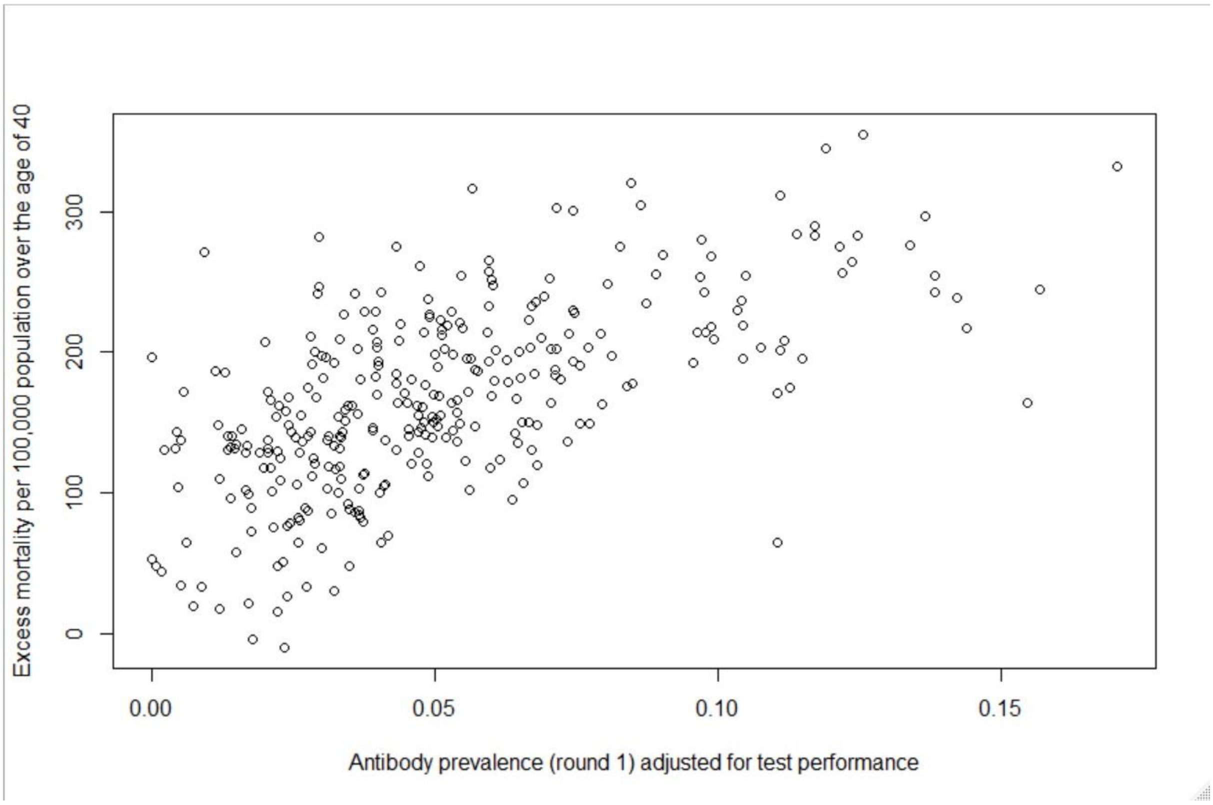


[1] Ward H, Atchison C, Whitaker M, Ainslie KEC, Elliott J, Okell L, Redd R, Ashby D, Donnelly C, Barclay W, Darzi A, Cooke G, Riley S, Elliott P. SARS-CoV-2 antibody prevalence in England following the first peak of the pandemic. *Nat Commun* 12, 905 (2021). https://doi.org/10.1038/s41467-021-21237-w

**Supplementary Fig. 4. Box plots with scatter showing how excess deaths varies with numbers of samples used in stage 1 and stage 2 of model**. Data are presented as scatter plots showing posterior mean excess mortality for MSOAs (n= 6,791 MSOAs) with box and whiskers plot superimposed showing, for excess mortality across MSOAs: median (centre line); interquartile range (IQR, 25th to 75th percentiles) (bounds of box); largest value no further than 1.5 * IQR from the box (top of upper whisker); and smallest value no further than 1.5*IQR from the box (bottom of lower whisker). (A) Excess mortality in males; (B) Excess mortality in females.

**A). Males**

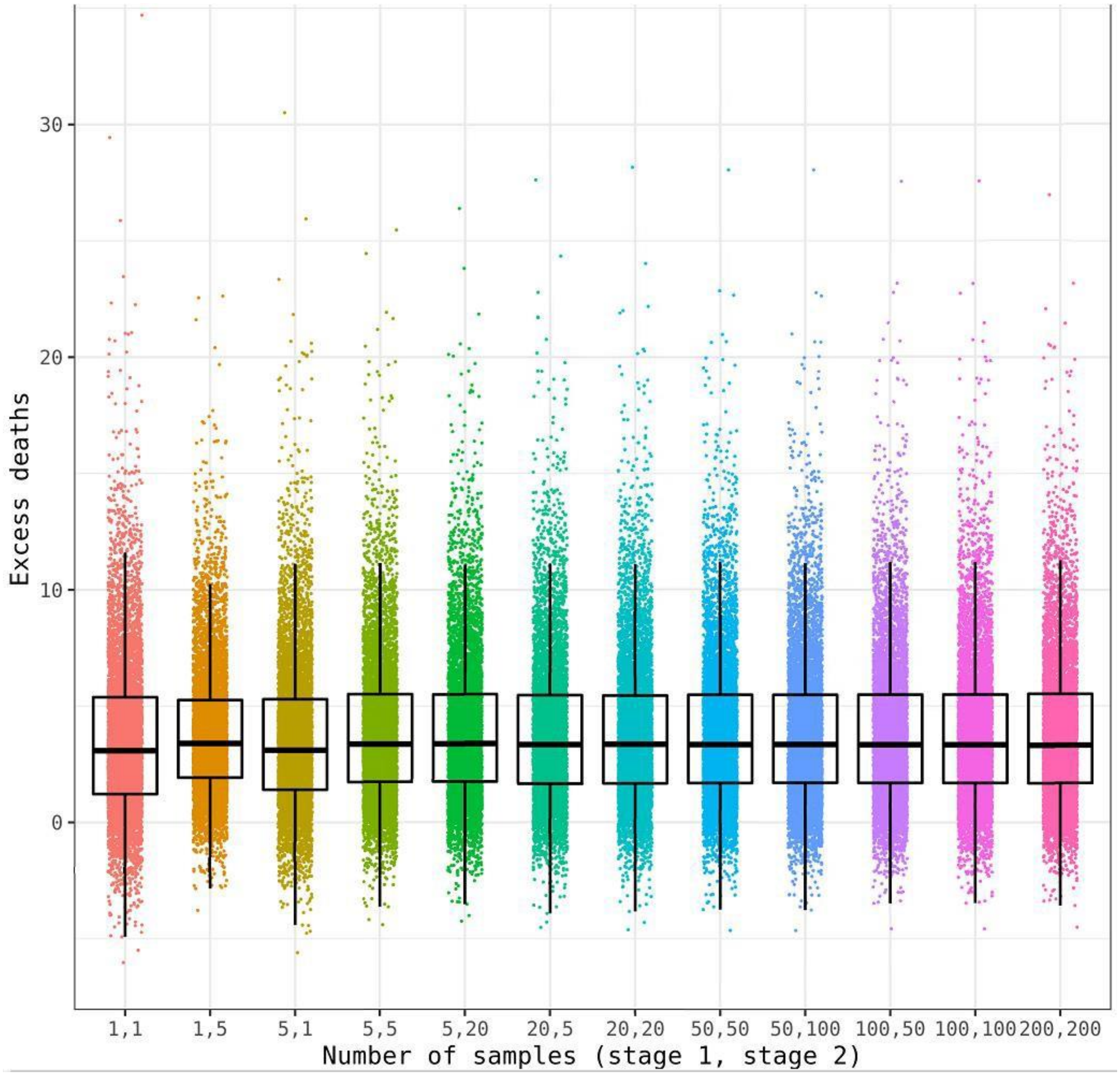

**B) Females**

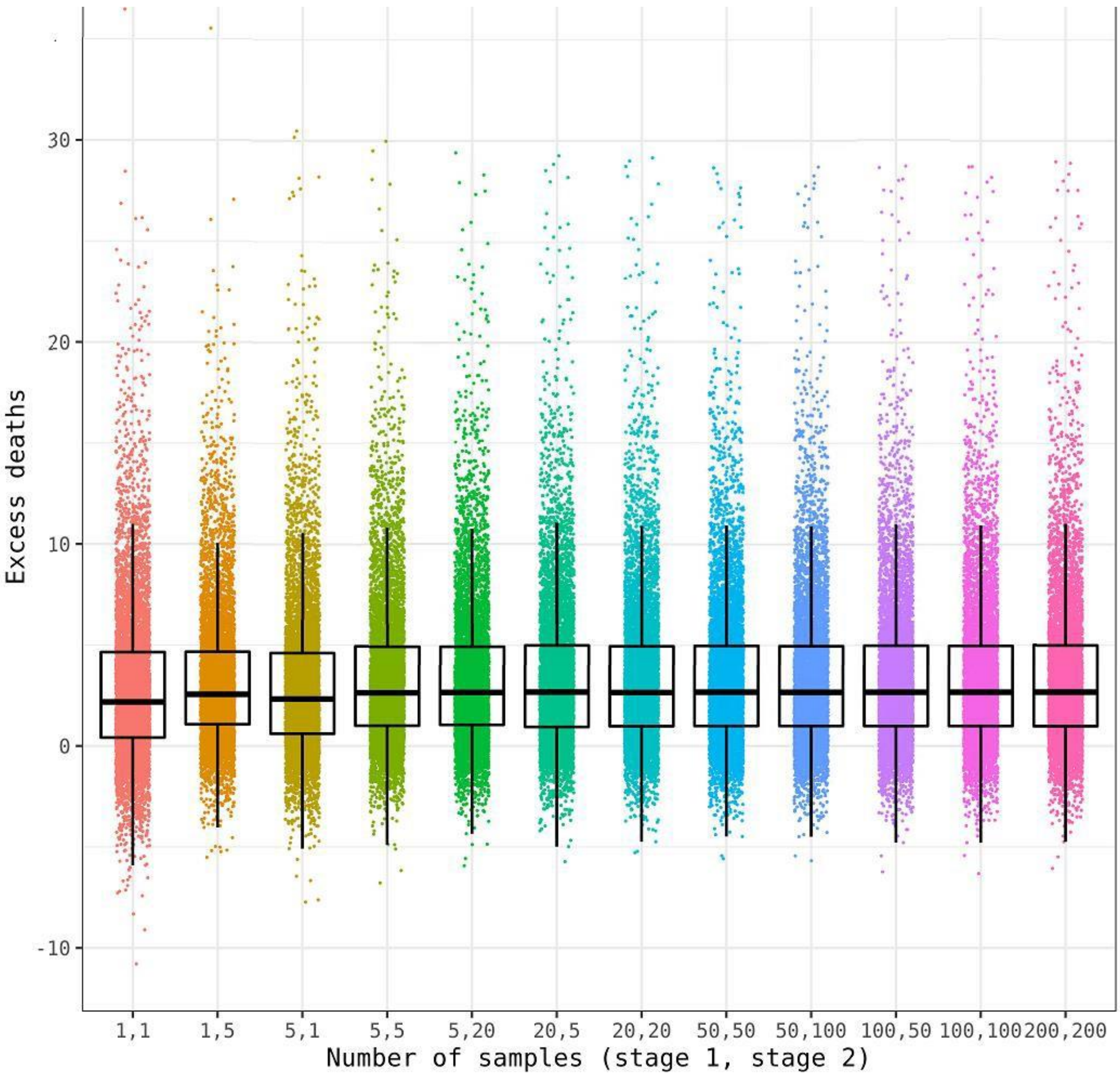
Excess deaths
30
20
10
0
-10
1,1
1,5
5,1
5,5
5,20
20,5
20,20
50,50
50,100
100,50
100,100
200,200
Number of samples (stage 1, stage 2)
