# Supplementary material for: Community factors and excess mortality in first wave of the COVID-19 pandemic in England
Source: Nat Commun. 2021 Jun 18;12:3755. doi: 10.1038/s41467-021-23935-x (PMC8213785; doi:10.1038/s41467-021-23935-x)
Supplement: Supplementary file 3 — Reporting summary [file 41467_2021_23935_MOESM3_ESM.pdf]

nature research

Corresponding author(s): Paul Elliott

Last updated by author(s): May 11, 2021

Reporting Summary

Nature Research wishes to improve the reproducibility of the work that we publish. This form provides structure for consistency and transparency in reporting. For further information on Nature Research policies, see our Editorial Policies and the Editorial Policy Checklist.

Statistics

For all statistical analyses, confirm that the following items are present in the figure legend, table legend, main text, or Methods section.

n/a	Confirmed	
☐	☒	The exact sample size (n) for each experimental group/condition, given as a discrete number and unit of measurement
☐	☒	A statement on whether measurements were taken from distinct samples or whether the same sample was measured repeatedly
☐	☒	The statistical test(s) used AND whether they are one- or two-sided<br>*Only common tests should be described solely by name; describe more complex techniques in the Methods section.*
☐	☒	A description of all covariates tested
☒	☐	A description of any assumptions or corrections, such as tests of normality and adjustment for multiple comparisons
☐	☒	A full description of the statistical parameters including central tendency (e.g. means) or other basic estimates (e.g. regression coefficient) AND variation (e.g. standard deviation) or associated estimates of uncertainty (e.g. confidence intervals)
☐	☒	For null hypothesis testing, the test statistic (e.g. F, t, r) with confidence intervals, effect sizes, degrees of freedom and P value noted<br>*Give P values as exact values whenever suitable.*
☐	☒	For Bayesian analysis, information on the choice of priors and Markov chain Monte Carlo settings
☐	☒	For hierarchical and complex designs, identification of the appropriate level for tests and full reporting of outcomes
☐	☒	Estimates of effect sizes (e.g. Cohen's d, Pearson's r), indicating how they were calculated

Our web collection on statistics for biologists contains articles on many of the points above.

Software and code

Policy information about availability of computer code

Data collection	No computer code was used.
Data analysis	DOI https://zenodo.org/record/4739256#.YJOvCC1Q0<br>Requires R software (https://www.r-project.org/) and R-INLA package (from https://www.r-inla.org/)

For manuscripts utilizing custom algorithms or software that are central to the research but not yet described in published literature, software must be made available to editors and reviewers. We strongly encourage code deposition in a community repository (e.g. GitHub). See the Nature Research guidelines for submitting code & software for further information.

Data

Policy information about availability of data

All manuscripts must include a data availability statement. This statement should provide the following information, where applicable:
- Accession codes, unique identifiers, or web links for publicly available datasets
- A list of figures that have associated raw data
- A description of any restrictions on data availability

• No identifiable information will be shared with any other organisation. SAHSU does not have permission to supply data to third parties. Individual mortality data can be requested through the Office for National Statistics (https://www.ons.gov.uk/).
• Mid-year population estimates can be downloaded from https://www.ons.gov.uk/peoplepopulationandcommunity/populationandmigration/populationestimates/datasets/middlesuperoutputareamidyearpopulationestimates.
• English Index of Multiple Deprivation data can be downloaded from https://www.gov.uk/government/statistics/english-indices-of-deprivation-2019.
• 2011 Census data can be downloaded from https://www.ons.gov.uk/census/2011census/2011censusdata.
• Modelled air pollution data (NO2 & PM2.5) can be downloaded from https://uk-air.defra.gov.uk/data/pcm-data.

• Locations data of care homes can be downloaded from https://covid19.esriuk.com/datasets/e4ffa672880a4facaab717dea3cdc404_0.
The results at MSOA level (excess deaths, credible intervals, posterior probabilities) used in figures 2 and 3 can be accessed at DOI: https://zenodo.org/record/4739256#.YJOvCC1Q0

Field-specific reporting

Please select the one below that is the best fit for your research. If you are not sure, read the appropriate sections before making your selection.

[x] Life sciences [ ] Behavioural & social sciences [ ] Ecological, evolutionary & environmental sciences

For a reference copy of the document with all sections, see nature.com/documents/nr-reporting-summary-flat.pdf

Life sciences study design

All studies must disclose on these points even when the disclosure is negative.

Sample size	All deaths in England in adults 40 years and over, 1 March to 31 May 2015-2020: 781,532 deaths.
Data exclusions	All deaths under age of 40. Deaths where age, sex or geocoding unknown.
Replication	As sensitivity analyses we re-ran the model using alternative priors for the hyperparameters τ_V and τ_U firstly using logGamma(0.5, 0.05), and secondly using the penalised complexity prior. Models run on Windows and Linux operating systems. Each model replicated 200 times to represent uncertainty, all replications successful.
Randomization	Not applicable in retrospective analysis of mortality at small-area level.
Blinding	Not applicable in retrospective analysis of mortality at small-area level.

Reporting for specific materials, systems and methods

We require information from authors about some types of materials, experimental systems and methods used in many studies. Here, indicate whether each material, system or method listed is relevant to your study. If you are not sure if a list item applies to your research, read the appropriate section before selecting a response.

Materials & experimental systems

n/a	Involved in the study	
[x]	[ ]	Antibodies
[x]	[ ]	Eukaryotic cell lines
[x]	[ ]	Palaeontology and archaeology
[x]	[ ]	Animals and other organisms
[ ]	[x]	Human research participants
[x]	[ ]	Clinical data
[x]	[ ]	Dual use research of concern

Methods

n/a	Involved in the study	
[x]	[ ]	ChIP-seq
[x]	[ ]	Flow cytometry
[x]	[ ]	MRI-based neuroimaging

Human research participants

Policy information about studies involving human research participants

Population characteristics	All deaths in adults over 40 years in England during study period. Study period (1 March 2020 – 31 May 2020), 88,092 male deaths, 86,235 female deaths. Comparison period (1 March – 31 May, 2015- 2019) 296,985 male deaths, 310,220 female deaths.
Recruitment	N/A, complete dataset of Death registrations
Ethics oversight	The study was covered by national research ethics approval from the London-South East Research Ethics Committee (Reference 17/LO/0846). Data access was covered by the Health Research Authority Confidentiality Advisory Group under section 251 of the National Health Service Act 2006 and the Health Service (Control of Patient Information) Regulations 2002 (Reference 20/CAG/0008).

Note that full information on the approval of the study protocol must also be provided in the manuscript.
